# The miR-98-3p/JAG1/Notch1 axis mediates the multigenerational inheritance of osteopenia caused by maternal dexamethasone exposure in female rat offspring

**DOI:** 10.1038/s12276-022-00743-x

**Published:** 2022-03-24

**Authors:** Hui Han, Hao Xiao, Zhixin Wu, Liang Liu, Ming Chen, Hanwen Gu, Hui Wang, Liaobin Chen

**Affiliations:** 1 Division of Joint Surgery and Sports Medicine, Department of Orthopedic Surgery, Zhongnan Hospital of Wuhan University, Wuhan, 430071 China; 2 Hubei Provincial Key Laboratory of Developmentally Originated Disease, Wuhan, 430071 China; 3 Department of Pharmacology, School of Basic Medical Sciences, Wuhan University, Wuhan, 430071 China

**Keywords:** Bone development, Reproductive disorders

## Abstract

As a synthetic glucocorticoid, dexamethasone is widely used to treat potential premature delivery and related diseases. Our previous studies have shown that prenatal dexamethasone exposure (PDE) can cause bone dysplasia and susceptibility to osteoporosis in female rat offspring. However, whether the effect of PDE on bone development can be extended to the third generation (F3 generation) and its multigenerational mechanism of inheritance have not been reported. In this study, we found that PDE delayed fetal bone development and reduced adult bone mass in female rat offspring of the F1 generation, and this effect of low bone mass caused by PDE even continued to the F2 and F3 generations. Furthermore, we found that PDE increases the expression of miR-98-3p but decreases JAG1/Notch1 signaling in the bone tissue of female fetal rats. Moreover, the expression changes of miR-98-3p/JAG1/Notch1 caused by PDE continued from the F1 to F3 adult offspring. Furthermore, the expression levels of miR-98-3p in oocytes of the F1 and F2 generations were increased. We also confirmed that dexamethasone upregulates the expression of miR-98-3p in vitro and shows targeted inhibition of JAG1/Notch1 signaling, leading to poor osteogenic differentiation of bone marrow mesenchymal stem cells. In conclusion, maternal dexamethasone exposure caused low bone mass in female rat offspring with a multigenerational inheritance effect, the mechanism of which is related to the inhibition of JAG1/Notch1 signaling caused by the continuous upregulation of miR-98-3p expression in bone tissues transmitted by F2 and F3 oocytes.

## Bone development: Dexamethasone increases multi-generational risk of low bone density

Research on rats indicates that the drug dexamethasone, commonly used to treat pregnant women at risk of premature birth, increases risks of impaired bone development that can be passed on through several generations. Liaobin Chen and colleagues at Wuhan University in China investigated the molecular mechanism behind dexamethasone-induced low bone density and the further inheritance of the condition. Exposing pregnant rats to dexamethasone reduced bone mass not only in the offspring of exposed mothers but also in two further generations. The research attributes the effect to alterations in identified gene regulation processes known to affect bone development, and suggests an epigenetic mechanism responsible for multi-generation inheritance. The results could spur the development of better treatments for women at risk of premature delivery, and patients with other conditions treated with dexamethasone.

## Introduction

Dexamethasone is a synthetic glucocorticoid that can easily cross the placental barrier to promote lung maturation in premature infants. Therefore, this drug is widely used in obstetric and pediatric diseases, especially in pregnant women at risk of preterm delivery^1,2^. However, more evidence has shown that prenatal synthetic glucocorticoid (such as dexamethasone) exposure results in intrauterine growth retardation of fetuses, leading to susceptibility to multiple diseases in adulthood^3–6^. Several studies have reported that the influence of glucocorticoid exposure during pregnancy on endocrine function and behavioral changes in offspring is not limited to current generations but can also be inherited over multiple generations^7^. Our recent studies also showed that prenatal dexamethasone exposure (PDE) causes developmental toxicity in the ovaries of offspring rats, which could be passed down to third generation (F3 generation) offspring^8,9^. Furthermore, the potential toxic effects of dexamethasone on offspring bone development during pregnancy have attracted extensive attention. Clinical studies have shown that the birth weight and body length of infants receiving dexamethasone treatment are lower than those of infants of the same gestational age; moreover, dexamethasone is an essential factor leading to a decrease in bone mineral content and bone mineral density after birth^10,11^. Animal studies also found that exposure to dexamethasone during the last 24 days of the fetal period in piglets can significantly decrease bone density and mass^12^. We previously confirmed that PDE has a toxic effect on the development of fetal bones, and this effect can continue into adulthood, causing susceptibility to osteoporosis^13,14^. However, it is not clear whether the effect of PDE on long bone mass in female offspring is sustainable in the F3 generation and its mechanism of multigenerational inheritance.

At present, it is believed that the mechanism of multigenerational inheritance is related to epigenetic modifications in somatic or germ cells^15,16^. MicroRNAs (miRNAs), as epigenetic mediators, usually bind to the 3’-untranslated regions (3’-UTRs) of target gene mRNAs to promote mRNA degradation or inhibit mRNA translation, thereby regulating gene expression at the post-transcriptional level. Furthermore, miRNAs are involved in the epigenetic regulation of multigenerational inheritance^17–19^. For example, it was found that changes in sperm miRNAs mediated the multigenerational inheritance of obesity and insulin resistance in offspring caused by a paternal high-fat diet^20^. Our recent findings demonstrated that the expression change of miR-320a-3p in oocytes mediates the multigenerational inheritance of inhibited ovarian estrogen synthesis induced by PDE^9^. These studies suggest that miRNAs may participate in the multigenerational inheritance of bone mass caused by PDE, although the regulatory mechanism is still unclear.

In this study, we established a rat offspring model induced by dexamethasone exposure during the middle and late pregnancy periods. The multigenerational inheritance phenomenon of bone mass induced by PDE in female offspring rats was investigated by detecting the changes in bone mass indices and functional genes of the long bone in the F1 to F3 generations. Furthermore, according to the miRNA sequencing analysis and the detection of miRNA expression in bone tissue and oocytes, we clarified the potential mechanism of the multigenerational inheritance of osteopenia caused by PDE. This study helps to reveal the long-term adverse effects of PDE on bone development and its early intervention targets and provides a theoretical basis for illuminating the multigenerational inheritance effect of adult diseases.

## Materials and methods

### Chemicals and reagents

Dexamethasone was obtained from Shuanghe Pharmaceutical Co., Ltd. (Wuhan, China). Human chorionic gonadotropin (hCG) was purchased from Shanghai Yiyan Biotechnology Co., Ltd. (Shanghai, China). The TRIzol reagent kit was obtained from Invitrogen (Carlsbad, CA, USA). mRNA reverse transcription and real-time quantitative polymerase chain reaction (RT–qPCR) kits were purchased from Vazyme Biotechnology Co., Ltd. (Nanjing, China). miRNA reverse transcription and RT–qPCR kits were purchased from Qiagen Biotechnology Co. (Qiagen, Germany). Some of the primers were synthesized by Tianyihuiyuan Biotechnology Co., Ltd. (Wuhan, China). The miRNA primers and miR-98-3p inhibitor or mimics were obtained from Guangzhou RiboBio Co., Ltd. (Guangzhou, China). Alizarin red dye was purchased from Yuanye Biotechnology Co., Ltd. (Shanghai, China). An alkaline phosphatase color development kit was purchased from Beyotime Biotechnology Co., Ltd. (Shanghai, China). Fetal bovine serum (FBS) and α-MEM were provided by Gibco (St Louis, MO, USA). The antibody for Jagged1 (JAG1) (sc-390177) was purchased from Santa Cruz Biotechnology, Inc. (Santa Cruz, CA, USA). The antibody for Notch1 (CST #4147) was purchased from Cell Signaling Technology, Inc. (Danvers, MA, USA). JAG1 overexpression plasmids were purchased from GeneChem Co., Ltd. (Shanghai, China). Other chemicals and reagents were of analytical grade.

### Animals and treatment

Specific pathogen-free Wistar rats (with weights of 200–240 g for females and 260–300 g for males) were purchased from the Experimental Center of the Hubei Medical Scientific Academy (No. 2017-0018, certification number: 42000600014526, Hubei, China). All animal experiments were performed in the Center for Animal Experiment of Wuhan University (Wuhan, China), which is accredited by the Association for Assessment and Accreditation of Laboratory Animal Care International (AAALAC International). The rats were housed in a temperature-controlled room (temperature: 18–22 °C; humidity: 40%–60%; light cycle: 12 h light-dark cycle) and allowed free access to food and water. After 1 week of adaptive feeding, the animal experiment was started. All animal experimental procedures were performed in accordance with the Guidelines for the Care and Use of Laboratory Animals of the Chinese Animal Welfare Committee.

Two 12-week-old female rats were placed together with one 12-week-old male rat overnight in a cage for mating. The next day, female rats were examined for vaginal smears. The presence of sperm in vaginal smears confirmed mating, and the mating date was designated gestational day (GD) 0. Pregnant rats were randomly divided into the control and PDE groups. From GD9 to GD20, the rats in the PDE group were subcutaneously injected with 0.2 mg/kg.d dexamethasone at 9:00 a.m. every day, and the rats in the control group were given saline at the same volume. Some pregnant rats were randomly selected from the two groups and sacrificed after administration of anesthesia with isoflurane at GD20 to obtain fetal rats of the F1 generation (*n* = 8 per group). Pregnant rats with litter sizes of 10 to 14 were considered qualified. The female fetuses were decapitated immediately to collect long bones. The left femurs and tibias were fixed with 4% paraformaldehyde overnight and embedded in paraffin for histological or immunohistochemistry analysis. The right femurs and tibias were stored in a refrigerator at −80 °C for further analysis. It should be noted that three whole fetal long bones in three different fetal rats from each litter were randomly counted as one sample and processed for gene analysis.

The rest of the pregnant rats, including those in the control and PDE groups, went into spontaneous labor to produce F1 adult offspring. Pregnant rats with a litter size of 10 to 14 were retained and then normalized to 10 (the male/female ratio was approximately 1:1). There were at least eight pregnant rats in each group. At postnatal week 8 (PW8), one female rat was randomly taken from each litter, anesthetized with isoflurane, and euthanized; then, the long bone tissue was rapidly collected. The left femurs and tibias were used for microcomputed tomography (micro-CT) analysis. The right femurs and tibias were used for subsequent analyses, including histological or immunohistochemistry analysis and gene expression analysis.

**Fig. 1:**
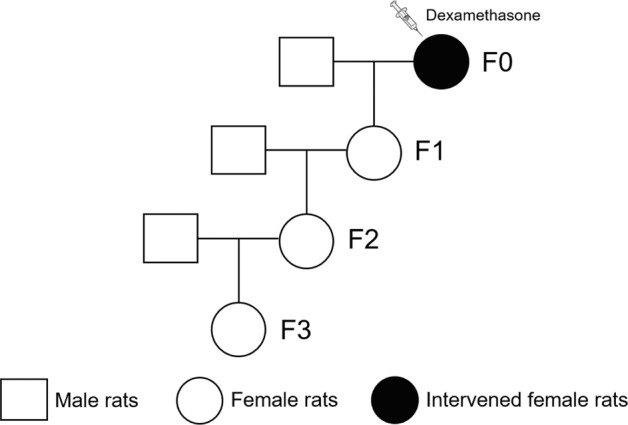
The animal experimental procedure. Prenatal dexamethasone exposure model and multigenerational phenotype via maternal-line.

The remaining F1 female offspring from the control and PDE groups in adulthood mated with normal male rats to generate F2 offspring. The F2 female offspring were culled using the same protocol as the F1 generation to consecutively produce F3 offspring. At PW8, the female F2 and F3 generations underwent studies similar to those performed on the F1 generation. The experimental procedures and treatment methods in this study are described as follows (Fig. 1).

### Isolation of oocytes

Pregnant mare serum gonadotropin (PMSG) hormones were intraperitoneally injected into the F1 and F2 female rats. After 24 h, the same dose of human chorionic gonadotropin (hCG) hormone was injected intraperitoneally. Fourteen to 16 h after hCG injection, the female rats were sacrificed after the administration of isoflurane anesthesia. The oviducts were dissected under a microscope, and oocytes were collected. The surrounding granulosa cells were removed, and the oocytes were stored in liquid nitrogen for subsequent detection.

### Histological and immunohistochemistry analysis

The femurs of fetal and adult rats were soaked in 4% paraformaldehyde overnight and then paraffin-embedded. Serial longitudinal section (5 µm thick) were cut. One out of every six sections was used for hematoxylin-eosin (H&E) staining to quantify the length of the primary ossification center. For Von Kossa staining, the sections were dewaxed and stained with 5% AgNO_3_ until they became dark brown. For immunohistochemical analysis, the sections were dewaxed and hydrated through a graded series of ethanol. Then, the sections were placed in 0.01 M sodium citrate buffer (pH 6.0) and boiled at approximately 95 °C for 10–15 min until antigen retrieval. After antigen retrieval, the hydrated sections were incubated in 3% H_2_O_2_ for 15 min to quench endogenous peroxidase activity. Sections were then blocked in 3% bovine serum albumin (BSA) (Servicebio, Wuhan, China) at room temperature for 1 h and incubated with primary antibodies against JAG1 (1:100 dilution) and Notch1 (1:200) overnight at 4 °C. After the sections were rinsed with PBS, they were incubated with biotinylated secondary antibody (1:100 dilution) and then incubated with avidin biotinylated horseradish peroxidase complex solution according to the manufacturer’s instructions. Finally, peroxidase activity was detected by immersion in diaminobenzidine (DAB) substrate. The staining intensity was determined by measuring the mean optical density (MOD) in six random fields for each section.

### Micro-CT scan

The obtained femur was immobilized with 70% ethanol. Then, the bone mass was scanned and analyzed with a VivaCT 40 μCT system (Scanco, Switzerland) as previously described^21^. To measure bone volume/total volume (BV/TV), bone trabecular number (Tb.N), bone trabecular thickness (Tb.Th), and bone trabecular separation (Tb.Sp), we selected 0.5–5.5 mm below the lowest point of the growth plate as the area of interest. The scanning resolution of the cross-sectional image was 21 μm. After scanning, three-dimensional reconstruction and quantitative analysis of cancellous bone were performed.

### Cell isolation, culture, and treatment

As previously described, bone marrow mesenchymal stem cells (BMSCs) were obtained from the femur and tibia of female Wistar rats at 3–4 weeks and cultured in complete growth medium (α-MEM with 10% FBS, 100 μg/ml streptomycin, and 100 U/ml penicillin)^22^. Then, we verified the multidirectional differentiation potential of BMSCs by osteogenic, chondrogenic and adipogenic differentiation (Supplementary Fig. 1). For induction of osteogenic differentiation, third-passage BMSCs were seeded in 6-well plates and treated with osteogenic induction medium (α-MEM with 10% FBS, 100 μg/ml streptomycin, 100 U/mL penicillin, 10 mM β-glycerophosphate, 50 μg/mL ascorbic acid, and 10 nM dexamethasone). Then, the cells were treated with various concentrations of dexamethasone or cotreated with dexamethasone and miRNA inhibitor or JAG1 overexpression plasmid for further analysis.

### Alkaline phosphatase and Alizarin red staining for BMSCs

Alkaline phosphatase (ALP) staining was performed using an Alkaline Phosphatase Color Development Kit according to the manufacturer’s instructions. For Alizarin red staining (ARS), the cells were washed twice with PBS and fixed with 4% paraformaldehyde for 10 min, rinsed with double-distilled H_2_O, and stained with 0.1% Alizarin red dye (pH 4.2) for 20 min at room temperature. Then, they were washed again with double-distilled H_2_O and observed by microscopy.

### Total RNA extraction and RT–qPCR

Total RNA was extracted with TRIzol reagent. The concentration and purity of total RNA were detected by a NanoDrop 2000 nucleic acid analyzer. For mRNA detection, cDNA was synthesized by using HiScript III RT SuperMix for qPCR (+gDNA wiper) (Vazyme) and then quantified by RT–qPCR with AceQ Universal SYBR qPCR Master Mix (Vazyme). For miRNA detection, cDNA was synthesized by using the miScript II RT Kit (Qiagen) and then quantified by RT–qPCR with SYBR Green PCR Master Mix (Qiagen). The primer sequences are all shown in Table 1. The relative expression of mRNA and miRNA was analyzed by the 2^–ΔΔCt^ method and normalized to the expression of GAPDH and U6, respectively.

**Table 1:** Primers used for real-time quantitative polymerase chain reaction.

Genes	Forward primers	Reverse primers	Annealing
Runx2	TACTTCGTCAGCGTCCTATC	CAGCGTCAACACCATCATT	60 °C, 30 s
Osterix	GGAGGCACAAAGAAGCCATA	GGGAAAGGGTGGGTAGTCAT	60 °C, 30 s
OCN	GGGCAGTAAGGTGGTGAATAG	CTAAACGGTGGTGCCATAGAT	60 °C, 30 s
COL1A1	AACAAGGTGACAGAGGCATAAA	GGCAGGAAGCTGAAGTCATAA	60 °C, 30 s
GAPDH	GGGTGTGAACCACGAGAAAT	ACTGTGGTCATGAGCCCTTC	60 °C, 30 s

*Runx2* Runt-related transcription factor 2; *OCN* Osteocalcin; *COL1A1* α1 chain of type I collagen gene; *GAPDH* Glyceraldehyde 3-phosphate dehydrogenase.

### MiRNA microarray analysis

Total RNA was isolated from fetal long bones at GD20 using Magzol Reagent (Magen, China) according to the manufacturer’s protocol. The quantity and integrity of the RNA yield were assessed by using K5500 (Beijing Kaiao, China) and Agilent 2200 TapeStation (Agilent Technologies, USA), respectively. Briefly, total RNA was ligated with a 3’ RNA adapter, followed by 5’ adapter ligation. Subsequently, the adapter-ligated RNAs were subjected to RT–PCR and amplified with a low cycle. Then, the PCR products were size selected on a PAGE gel according to the NEBNext Multiplex Small RNA Library Prep Set for Illumina (Illumina, USA). Finally, the purified library products were evaluated using an Agilent 2200 TapeStation system and Qubit (Thermo Fisher Scientific, USA). The libraries were sequenced on an Illumina HiSeq 2500 system (Illumina, USA) with a single-end 50 bp sequence at RiboBio Co., Ltd.

### Western blot analysis

The cells were washed with PBS and then lysed on ice with RIPA lysis buffer (Beyotime, Shanghai, China) containing 1 mM PMSF and protease inhibitor cocktail for 30 min to extract the total protein. A BCA protein assay kit (Beyotime, Shanghai, China) was used to detect the protein concentration of the samples. A total of 30 μg of protein was loaded into each lane, isolated by sodium dodecyl sulfate–polyacrylamide gel electrophoresis (SDS–PAGE) (10% gels), and then transferred to polyvinylidene difluoride membranes. The membranes were blocked with 5% skim milk at room temperature for 1 h and then incubated overnight at 4 °C with specific antibodies: JAG1 (1:200) and Notch1 (1:1000). On the second day, the membranes were incubated with horseradish peroxidase-conjugated secondary antibody at room temperature for 1 h. Finally, the protein bands were visualized by using chemiluminescent ECL reagent. The bands were quantified by measuring the mean optical density for each group.

### Dual luciferase reporter system luciferase assay

The JAG1 3’-UTR containing the conserved miR-98-3p binding sites or corresponding mutated sites was synthesized by GeneChem (Shanghai, China) and amplified by PCR. The PCR fragment was subcloned into the SacI and HindIII sites downstream of the luciferase reporter gene in the psiCHECK™-2 vector (Promega, Madison, USA). The luciferase reporter vector was cotransfected with miR-98-3p mimic or miR-negative control (miR-NC) into HEK-293T cells using Lipofectamine 3000 (Invitrogen). The luciferase activities were measured 48 h after transfection using the Dual Luciferase Reporter Assay System (Promega) according to the manufacturer’s instructions. The Renilla luciferase activity was normalized to the firefly luciferase activity for each transfected well.

### Statistical analysis

The data were analyzed and graphed by SPSS and GraphPad Prism 7 software. All of the numerical results are presented as the mean ± standard error of the mean (S.E.M.). Significant differences between the control and treatment groups were identified using Student’s *t* tests. The differences among more than two groups were determined using one-way analysis of variance (ANOVA). *P* < 0.05 was considered statistically significant.

## Results

### PDE delayed fetal bone development in female rats of the F1 generation

**Fig. 2:**
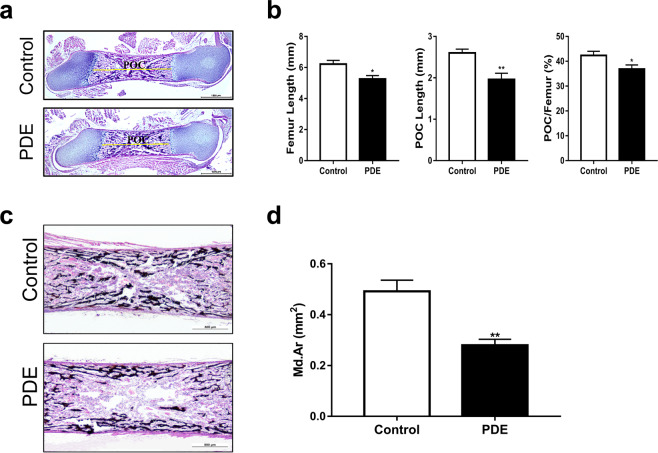
Effect of PDE on long bone development in female fetal rats. **a** Representative H&E staining images of fetal rat femurs (scale bar = 1000 μm). **b** Quantitative analysis of femur length, primary ossification center length, and primary ossification center length/total length of the femur in fetal rats. **c** Representative Von Kossa staining images of fetal rat femurs (scale bar = 500 μm). **d** Quantitative analysis of the mineralization zone in the primary ossification center. PDE Prenatal dexamethasone exposure; Md. Ar Mineralized area. Mean ± S.E.M., *n* = 8. ^*^P < 0.05, ^**^P < 0.01 *vs*. the control.

First, we observed the effects of PDE on fetal bone development. The H&E staining results showed that PDE significantly shortened the entire femur length and the length of the primary ossification center of the femur compared with the control (*P* < 0.05, *P* < 0.01, Fig. 2a, b). Moreover, we analyzed the calcification of the primary ossification center by Von Kossa staining and found that PDE significantly reduced the mineralization area of the primary ossification center in F1 generation female fetal rats (*P* < 0.01, Fig. 2c, d). These results suggested that PDE could delay bone development and reduce bone mineralization in female fetal rats of the F1 generation.

### PDE reduced bone mass in adult female rats of the F1-F3 generations

**Fig. 3:**
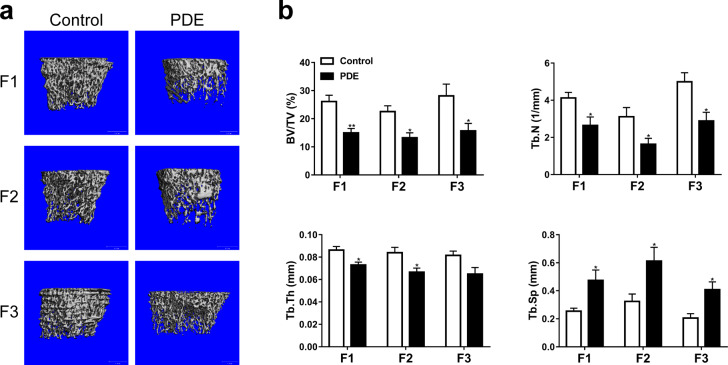
Effect of PDE on postnatal long bone mass in F1-F3 female rats. **a** Representative micro-CT images of the femur in 8-week-old rats of the F1-F3 generations, scale bar = 1000 μm. **b** Quantitative micro-CT analysis of trabecular bone microarchitecture. PDE Prenatal dexamethasone exposure; BV/TV Bone volume/trabecular volume; Tb.N Trabecular number; Tb.Th Trabecular thickness; Tb.Sp Trabecular separation. Mean ± S.E.M., *n* = 8. ^*^P < 0.05, ^**^P < 0.01 *vs*. the control.

To investigate whether the effect of PDE on the bone mass formation of F1 female offspring can continue to adulthood or even the F3 generation, we detected the bone mass of 8-week-old female offspring of the F1-F3 generations by micro-CT. The results showed that PDE significantly reduced the bone mass of femoral cancellous bone of the F1- F3 generations, which was characterized by decreased BV/TV, Tb.N, and Tb.Th and increased Tb.Sp (*P* < 0.05, *P* < 0.01, Fig. 3a, b). These results suggested that PDE reduced bone mass in female rat offspring and that there was a multigenerational inheritance phenomenon.

### PDE increased miR-98-3p expression in bone tissues of F1-F3-generation female rats through oocytes

**Fig. 4:**
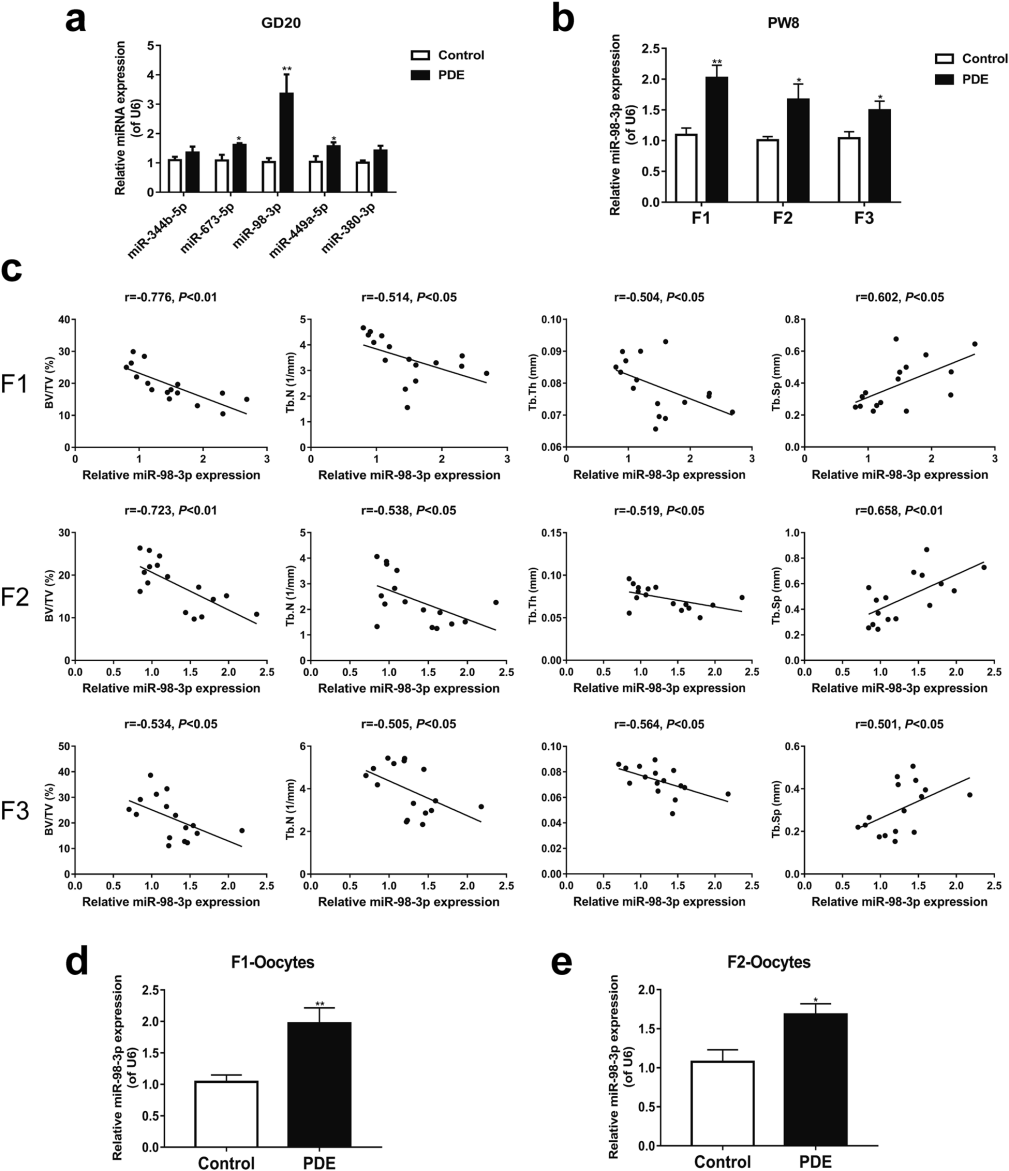
Effect of PDE on miR-98-3p expression in bone tissue and oocytes of female rats. **a** RT-qPCR was used to validate candidate miRNAs during the intrauterine period (*n* = 8). **b** Expression level of miR-98-3p in long bone tissues of the F1-F3 adult rats (*n* = 8). **c** Correlation analysis between the relative expression of miR-98-3p and BV/TV, Tb.N, Tb.Th, and Tb.Sp in F1-F3-generation long bone. **d**, **e** Expression changes of miR-98-3p in F1 and F2-generation oocytes (*n* = 6). GD Gestational day; PW Postnatal week; PDE Prenatal dexamethasone exposure; BV/TV Bone volume/trabecula volume; Tb.N Trabecula number; Tb.Th Trabecula thickness; Tb.Sp Trabecular separation. Mean ± S.E.M., ^*^P < 0.05, ^**^P < 0.01 *vs*. the control.

Next, we explored the multigenerational inheritance mechanism of PDE-induced bone dysplasia and low bone mass in female rat offspring. We performed a miRNA microarray analysis on female fetal rat bone tissue of the F1 generation from the control and PDE groups (Supplementary Fig. 2a). RT–qPCR was further used to verify the five candidate miRNAs with the most apparent expression differences. The results showed that PDE significantly increased the expression of miR-98-3p in the bone tissue of the F1 female fetal rats (*P* < 0.01, Fig. 4a), and increased expression of miR-98-3p continued after birth and in the F2 and F3 generations (*P* < 0.05, *P* < 0.01, Fig. 4b). However, other miRNAs (e.g., miR-673-5p or miR-449a-5p) with significant changes during the intrauterine period could not be stably inherited by the F3 generation (Supplementary Fig. 2b). The correlation analysis results indicated that the expression of miR-98-3p in bone tissue of the F1-F3 generations was significantly correlated with bone mass index (*P* < 0.05, *P* < 0.01, Fig. 4c). These results suggested that PDE induced osteopenia in F1-F3 female offspring rats, which was related to the increased expression of miR-98-3p in bone tissue.

Furthermore, we compared the mature sequences of miR-98-3p in multiple species. Sequence analysis of miR-98-3p revealed that it is highly conserved in *Homo sapiens*, *Mus musculus*, *Rattus norvegicus*, and other species (Supplementary Fig. 3a). To clarify whether the continuous increase in miR-98-3p expression in the bone tissues of the F1-F3 offspring induced by PDE was transmitted through oocytes, we detected the expression of miR-98-3p in the oocytes of the F1 and F2 generations. The results showed that the expression of miR-98-3p in F1 and F2 oocytes of the PDE group was also higher than that of the control group (*P* < 0.05, *P* < 0.01, Fig. 4d, e). Collectively, the above results revealed that PDE could increase the expression of miR-98-3p in the bone tissue of F1 female rats and transmit it to the F2 and F3 generations *via* oocytes, thereby mediating the occurrence of a multigenerational inheritance effect of osteopenia.

### Upregulation of miR-98-3p expression mediated the inhibition of osteogenic differentiation induced by PDE in the F1-F3 female rats

**Fig. 5:**
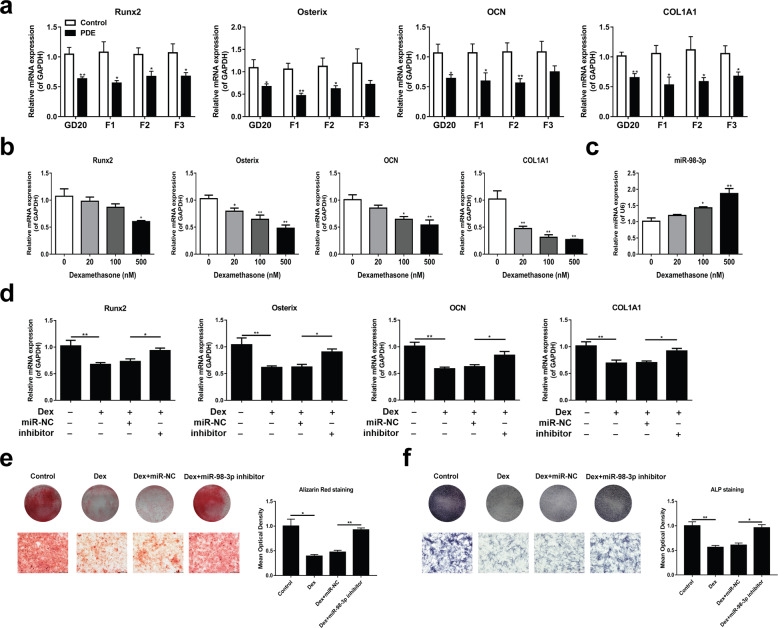
miR-98-3p mediated PDE-induced inhibition of osteogenic differentiation in F1-F3 female rats. **a** mRNA expression of femoral osteogenic differentiation marker genes in the F1 fetal rats and the F1-F3 adult rats (*n* = 8). **b** RT-qPCR was used to detect the expression of osteogenic differentiation marker genes in the BMSCs treated with different concentrations of dexamethasone during osteogenic differentiation (*n* = 3). **c** RT-qPCR was used to detect the expression of miR-98-3p in the BMSCs treated with different concentrations of dexamethasone (*n* = 3). **d** RT-qPCR was used to analyze the expression of osteogenic differentiation marker genes in the BMSCs transfected with miR-98-3p inhibitor under 500 nM dexamethasone and osteogenic differentiation conditions (*n* = 3). **e** Mineral deposition was indicated by Alizarin red staining (*n* = 3). **f** ALP staining was performed on Day 7 of osteogenic differentiation (*n* = 3). GD Gestational day; PDE Prenatal dexamethasone exposure; Runx2 Runt-related transcription factor 2; OCN Osteocalcin; COL1A1, α1 chain of type I collagen gene; Dex Dexamethasone; miR-NC miRNA negative control. Mean ± S.E.M., ^*^P < 0.05, ^**^P < 0.01 *vs*. the appropriate controls.

Osteogenic differentiation is an important factor affecting bone development and bone mass formation^23^. Therefore, we further detected the expression of femoral osteogenic differentiation marker genes (*Runx2*, *Osterix*, *Ocn*, and *Col1a1*) in F1 fetal rats and 8-week-old rats of the F1-F3 generations. The results showed that the expression of osteogenic differentiation marker genes in F1 fetal rats and F1-F3 female adult rats in the PDE group was significantly decreased (*P* < 0.05, *P* < 0.01, Fig. 5a). This result suggested that PDE could induce continuous inhibition of osteogenic differentiation in F1-F3 female rats.

Next, we verified the role of miR-98-3p in dexamethasone-induced inhibition of osteogenic differentiation of BMSCs. First, BMSCs were treated with different concentrations of dexamethasone (0, 20, 100, and 500 nM) in osteogenic differentiation culture, and then, the expression of osteogenic differentiation marker genes was detected. We found that dexamethasone inhibited the expression of osteogenic differentiation marker genes in a concentration-dependent manner and simultaneously upregulated the expression of miR-98-3p (*P* < 0.05, *P* < 0.01, Fig. 5b, c). Subsequently, we observed the effect of high-concentration (500 nM) dexamethasone on the osteogenic differentiation of BMSCs transfected with miR-98-3p inhibitor. RT–qPCR results showed that the miR-98-3p inhibitor alleviated the inhibitory effect of dexamethasone on osteogenic differentiation (*P* < 0.05, *P* < 0.01, Fig. 5d). Similar results were also obtained by ARS staining and ALP staining (Fig. 5e, f). In contrast, when we transfected the miR-98-3p mimic as a positive control, we found that the miR-98-3p mimic could further enhance the osteogenic inhibitory effect of dexamethasone (Supplementary Fig. 4). These findings indicated that miR-98-3p is involved in the inhibition of osteogenic differentiation in F1-F3 female rats induced by PDE (dexamethasone).

### miR-98-3p participated in dexamethasone-induced inhibition of osteogenic differentiation by targeting the decrease in JAG1/Notch1 signaling in F1-F3 female rats

**Fig. 6:**
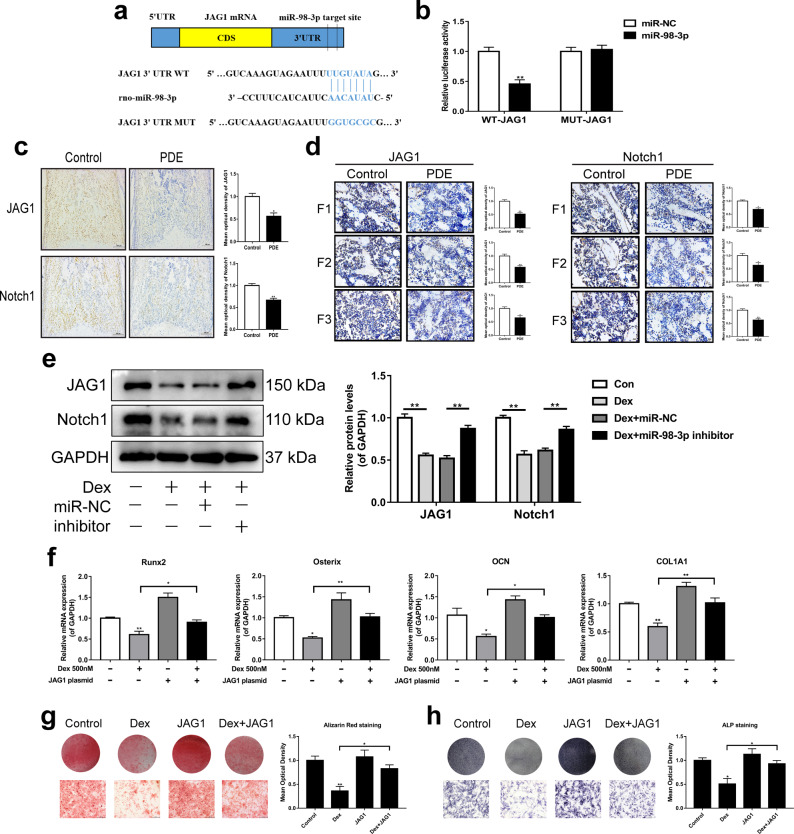
miR-98-3p participates in PDE-induced inhibition of osteogenic differentiation in the F1-F3 generation by targeting the decrease in JAG1/Notch1 signaling. **a** Schematic representation of a predicted binding site of miR-98-3p in the 3’-UTR of JAG1 mRNA and the mutant JAG1 3’-UTR. **b** The luciferase activity was determined using the Dual Luciferase Reporter System (*n* = 3). **c** Immunohistochemistry and MOD analysis of JAG1 and Notch1 in the primary ossification center of fetal rats (*n* = 5), scale bar = 100 μm. **d** Immunohistochemistry and MOD analysis of femur JAG1 and Notch1 in 8-week-old rats of the F1-F3 generations (*n* = 5), scale bar = 10 μm. **e** Western blot analysis of JAG1/Notch1 expression in the BMSCs treated with 500 nM dexamethasone and miR-98-3p inhibitor (*n* = 3). **f** RT–qPCR analysis of the expression of osteogenic marker genes in the BMSCs treated with 500 nM dexamethasone and the JAG1 plasmid (*n* = 3). **g** Mineral deposition was indicated by Alizarin red staining (*n* = 3). **h** ALP staining was performed on Day 7 of osteogenic differentiation (*n* = 3). JAG1 Jagged1; Con control; MOD mean optical density; miR-NC miRNA negative control; PDE prenatal dexamethasone exposure; Dex dexamethasone; Runx2 Runt-related transcription factor 2; OCN osteocalcin; COL1A1 α1 chain of type I collagen gene. Mean ± S.E.M., ^*^P < 0.05, ^**^P < 0.01 *vs*. the control.

To gain insights into the molecular mechanisms by which miR-98-3p regulates the osteogenic differentiation of BMSCs, we predicted the potential targets of miR-98-3p using bioinformatics tools (TargetScan) and found that the 3’-UTR of JAG1, a key ligand of the Notch signaling pathway, has a miR-98-3p binding site. Moreover, the binding site is highly conserved among vertebrates (Supplementary Fig. 3b). We next constructed luciferase reporters that had either a wild-type (WT) 3’-UTR or a 3’-UTR containing mutant sequences of the miR-98-3p binding site to confirm that miR-98-3p can directly target JAG1 (Fig. 6a). The results (Fig. 6b) showed that overexpression of miR-98-3p remarkably inhibited the luciferase reporter activity of the WT JAG1 3’-UTR (*P* < 0.01) but not that of the mutated 3’-UTR. These results indicated that miR-98-3p could directly regulate the expression of JAG1.

Previous studies have suggested that JAG1 can activate the Notch signaling pathway by affecting Notch1 and then participate in the regulation of osteogenic differentiation^24,25^. Then, we detected the protein expression of JAG1 and Notch1 by immunohistochemistry. The results showed that the expression of JAG1 and Notch1 was significantly decreased in the PDE group from the intrauterine period of the F1 generation to adulthood as well as in the subsequent F2 and F3 generations (*P* < 0.05, *P* < 0.01, Fig. 6c, d). In addition, Western blot analyses indicated that dexamethasone could significantly reduce the expression of JAG1 and Notch1 in BMSCs, and the miR-98-3p inhibitor could partially reverse the inhibition of JAG1 and Notch1 expression induced by dexamethasone (*P* < 0.01, Fig. 6e). Furthermore, we explored the role of JAG1/Notch1 signaling in inhibiting the osteogenic differentiation of BMSCs by dexamethasone in vitro. We found that overexpression of JAG1 alleviated the inhibitory effect of dexamethasone on the expression of osteogenic differentiation marker genes (*P* < 0.05, *P* < 0.01, Fig. 6f). This finding was consistent with the results of ARS and ALP staining (Fig. 6g, h). Taken together, these data demonstrated that miR-98-3p could participate in dexamethasone-induced inhibition of osteogenic differentiation by decreasing JAG1/Notch1 signaling.

## Discussion

### PDE caused the multigenerational inheritance of osteopenia in female offspring

The classic clinical application of dexamethasone in the treatment of premature infants is an intramuscular injection of 6 mg per 12 h for a total of four times over 24–34 weeks of gestation. A repeat course should be considered in women at risk of preterm birth 7 or more days after an initial course in women who remain at risk of preterm birth at less than 34 weeks of gestation^26,27^. To simulate the role of dexamethasone in the treatment of threatened preterm labor in the clinic, we administered dexamethasone to Wistar rats in the second and third trimesters of pregnancy. Based on the dose conversion relationship between rats and humans (conversion coefficient is 6.16:1)^28^, the dexamethasone administration dose of 0.2 mg/kg.d in rats in this study was comparable to the therapeutic dose of 0.03 mg/kg.d in humans. Since the standard dose for the clinical use of dexamethasone is 0.05-0.2 mg/kg.d^29^, the dose of dexamethasone administered in this study can be achieved in clinical practice. Moreover, due to the difficulty in the early diagnosis of preterm birth and the poor effect of some pregnant women with a single course of treatment, approximately 1/3 of pregnant women were changed from prophylactic drug administration to continuous drug administration^30^. Therefore, dexamethasone was given continuously at GD9-20 (the second and third trimesters of pregnancy) in this study. An epidemiological survey found that the incidence rate of osteoporosis in women over 45 years old is higher than that in men, accounting for 62%~83% of the total number of patients^31^. Some surveys around the world also found that the probability of osteoporotic hip fracture in older women is higher than that in men^32,33^. This result suggested that women are more likely to suffer from osteoporosis and have more typical symptoms. Therefore, the selection of female rats as the research object is representative.

Transgenerational inheritance refers to the phenomenon in which epigenetic markers and phenotypes can still be transmitted to offspring without direct environmental exposure^34^. The programming intervention of the mother (F0 generation) during pregnancy directly affects the development of her offspring in utero (F1 generation), and the germ cells (future gametes) that form the F2 generation are also directly exposed to the adverse environment during this pregnancy, while the F3 generation is not directly exposed. Therefore, only the effect on the F3 generation and above can be truly called transgenerational inheritance^35^. Studies have shown that an adverse environment during pregnancy can lead to transgenerational inheritance effects of various disease phenotypes. For example, a low-protein diet during pregnancy can lead to abnormal pancreatic development and glucose metabolism in offspring and has transgenerational inheritance effects^36^. In addition, zinc deficiency during pregnancy can lead to immunosuppression of offspring and be inherited by the F3 generation^37^. In a rat model of PDE, we observed that PDE can lead to low peak bone mass in male offspring, which can be inherited by the F2 generation^38^. On this basis, we further studied the effect of PDE on the bone mass of the F3 female generation and explored the possible mechanism of transgenerational inheritance. First, we found that PDE can significantly shorten the absolute length of the primary ossification center of the fetal long bones, result in sparse bone trabeculae, and reduce the expression of osteogenic marker genes. The bone mass of long bones and the expression of osteogenic differentiation marker genes were also significantly reduced at 8 postnatal weeks. This result suggested that the long bone dysplasia of female rat offspring induced by PDE has an intrauterine programming effect, which could last until after birth. Furthermore, we found that the long bone mass and osteogenic differentiation marker gene expression in the F2 and F3 generations of the PDE group were significantly reduced. This finding indicated that the inhibitory effect of PDE on bone development could continue through the maternal line to adulthood of the F2 and F3 generations and had a multigenerational inheritance effect.

### miR-98-3p mediated the multigenerational inheritance of PDE-induced osteopenia in female offspring through germ cells

Studies have shown that epigenetic modifications may be related to the multigenerational inheritance of some diseases^39^. Adverse prenatal environmental exposure often leads to abnormal epigenetic modification, which in turn leads to the occurrence of adverse phenotypes in offspring^40,41^. The main action sites of these different environmental factors are usually in the germline to promote the continuous inheritance of epigenetic modifications to the next generation, thereby providing a theoretical basis for the occurrence of some transgenerational diseases^42,43^. For example, it was reported that the offspring and even grandchildren of pregnant women with diabetes exhibit similar metabolic syndrome phenotypes, which may be related to epigenetic modifications of oocytes^44^. As a highly conserved epigenetic modification, miRNA plays a crucial role in regulating gene expression and genomic stability and may be involved in the occurrence of multigenerational inheritance phenomena^15^. For example, the injection of miR-1 into mouse fertilized eggs resulted in the development of a cardiac hypertrophy disease phenotype that could be inherited for at least three generations, and all three generations of spermatozoa showed persistent elevation of miR-1^45^. In this study, we verified that the expression of miR-98-3p in the long bone of the F1 generation before and after birth and the F2-F3 generation was significantly increased, as shown by high-throughput sequencing. We also confirmed that miR-98-3p is highly conserved. Moreover, we detected the expression of miR-98-3p in F1- and F2-generation oocytes and found that the expression of miR-98-3p was still elevated in PDE-group oocytes. These results suggested that the multigenerational inheritance effects of PDE-induced osteopenia in female offspring are due to the inheritance of F1- and F2-generation oocytes. The increased expression of miR-98-3p in adult long bone tissues of the F3 generation is maintained by stable mitosis of somatic cells derived from germ cells. The changes in miR-98-3p expression were retained in both germ cells and somatic cells, which together mediated the multigenerational inheritance of PDE-induced osteopenia in female offspring. Fetal-originated adult diseases usually have sex differences^46,47^; therefore, we also investigated the changes in bone mass and the expression of miR-98-3p in male rats. We found that PDE still caused low bone mass in male F3 rats but did not affect the expression of miR-98-3p in male F1 fetal rats (Supplementary Fig. 5). This finding indicated that there may be other mechanisms involved in the multigenerational inheritance of osteopenia in male rats; however, further research is needed.

Epigenetic markers undergo two rounds of reprogramming during the life cycle, one during gamete formation and one during fertilization, which is likely to result in the loss of genome-wide epigenetic information. However, reprogramming is not carried out thoroughly, and there may be epigenetic markers that are not completely cleared at each stage^48^. If environmental factors lead to permanent changes in the epigenome of parental germ cells, the transgenerational epigenetic inheritance will occur through the transmission of germ cells^49^. In this study, the increased expression of miR-98-3p in oocytes induced by PDE was sustainably inherited, possibly due to escape during reprogramming, thus preserving the abnormal changes in miR-98-3p. However, the detailed mechanism of epigenetic modification changes and how epigenetic modification in oocytes is stably transmitted to the next generation are not fully understood. Whether a deeper mechanism is involved, such as miRNA methylation and imprinted genes, remains to be further studied. In addition, this study lacks in vivo miR-98-3p inhibition experiments in oocytes, which will be the focus of our future research.

### miR-98-3p/JAG1/Notch1 signaling contributed to the multigenerational inheritance of PDE-induced osteopenia in female offspring

As a common epigenetic mediator, miRNAs typically regulate gene expression at the post-transcriptional level by promoting mRNA degradation or inhibiting mRNA translation through binding to the 3’-UTR of the target mRNAs^19,50^. In this study, we first screened the changes in the miRNA expression profile in fetal rat long bones induced by PDE by miRNA microarray analysis and confirmed that miR-98-3p could participate in the multigenerational inheritance of PDE-induced osteopenia through oocyte transmission. We further studied the mechanism at the cellular level and found that dexamethasone could increase the expression of miR-98-3p and inhibit the osteogenic differentiation of BMSCs in a concentration-dependent manner. The dexamethasone-mediated inhibition of osteogenic differentiation of BMSCs was partially reversed or enhanced by miR-98-3p inhibitor or mimics. This finding indicated that miR-98-3p could directly participate in and regulate the dexamethasone-mediated inhibition of osteogenic differentiation of BMSCs. We also confirmed that dexamethasone could regulate miR-98-3p by activating glucocorticoid receptors (Supplementary Fig. 6).

Furthermore, we discussed the downstream signaling pathway of miR-98-3p. JAG1 is one of the crucial ligands of the Notch signaling pathway. Interacting with Notch1 receptors leads to the release of the Notch intracellular domain (NICD), allowing it to translocate into the nucleus and activate Notch-responsive genes that are important for cell differentiation and morphogenesis in different biological systems^51,52^. JAG1/Notch1 signaling is also considered an essential factor in maintaining skeletal development and homeostasis in humans. A microdeletion of the JAG1 gene can cause a disease characterized by bone abnormalities (Alagille syndrome)^53^. Moreover, JAG1 has been shown to promote osteogenic differentiation of ligamentum flavum cells and human bone marrow mesenchymal stem cells through activation of Notch signaling^54,55^. In this study, we predicted that JAG1 was the target gene of miR-98-3p through bioinformatics and further confirmed it by dual luciferase reporter assays. Furthermore, in the multigenerational inheritance model of PDE, the expression levels of JAG1 and Notch1 continued to decrease. Cell experiments also confirmed that dexamethasone could increase miR-98-3p expression and decrease the expression of JAG1 and Notch1, while a miR-98-3p inhibitor could reduce the suppressive effects of dexamethasone on JAG1 and Notch1 expression. In addition, overexpression of JAG1 can partly rescue the inhibitory effect of dexamethasone on the osteogenic differentiation of BMSCs. These findings demonstrated that miR-98-3p and its molecular target JAG1/Notch1 signaling are involved in the multigenerational inheritance of PDE-induced osteopenia in offspring.

**Fig. 7:**
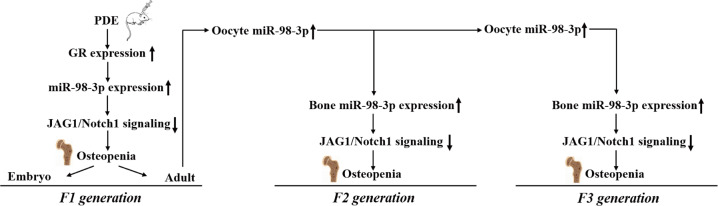
miR-98-3p/JAG1/Notch1 signaling mediates the multigenerational inheritance effect of osteopenia caused by maternal dexamethasone exposure in female rat offspring. PDE prenatal dexamethasone exposure; GR glucocorticoid receptor; JAG1 Jagged1.

In summary, this study confirmed that PDE caused the multigenerational inheritance effect of osteopenia in female offspring rats. The mechanism is related to the continuous upregulation of miR-98-3p expression induced by PDE in bone tissue transmitted through oocytes and then targeted inhibition of JAG1/Notch1 signaling, leading to poor osteogenic differentiation (Fig. 7). This study provides new experimental evidence for the analysis of bone developmental toxicity and multigenerational inheritance effects caused by PDE and a theoretical and experimental basis for exploring early treatment measures.

## Supplementary information

Supplementary materials
