# Supplementary material for: The miR-98-3p/JAG1/Notch1 axis mediates the multigenerational inheritance of osteopenia caused by maternal dexamethasone exposure in female rat offspring
Source: Exp Mol Med. 2022 Mar 24;54(3):298–308. doi: 10.1038/s12276-022-00743-x (PMC8979986; doi:10.1038/s12276-022-00743-x)
Supplement: Supplementary file 1 — Supplementary materials [file 12276_2022_743_MOESM1_ESM.pdf]

Supplementary data:

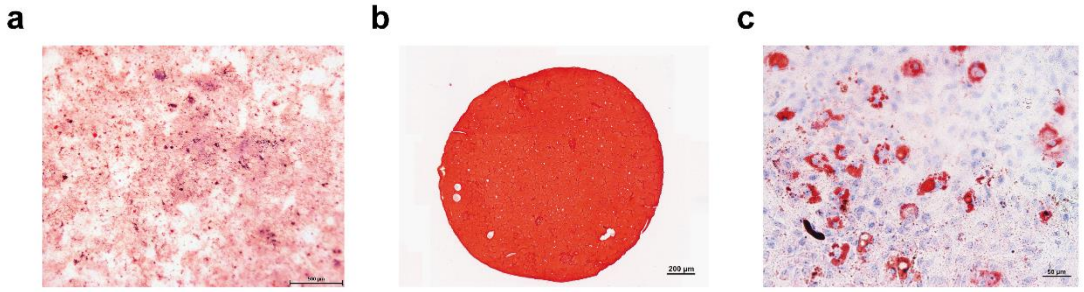

Supplementary Fig. 1 Three lineages differentiation of BMSCs. a. Alizarin red staining (osteogenesis differentiation, scale bar=500 μm). **b.** Safranin O staining (chondrogenic differentiation, scale bar=200 μm). **c.** Oil red O staining (adipogenic differentiation, scale bar=50 μm).

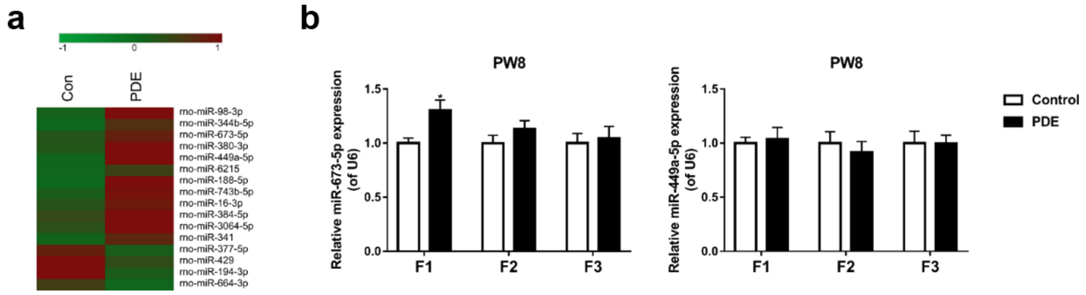

Supplementary Fig. 2 Expression pattern of miRNA in intrauterine period and adult F1-F3 generations of rats. a. MiRNA microarray was performed with total RNA isolated from primary ossification center of fetal rats. **b.** Expression level of miR-673-5p and miR-449a-5p in F1-F3 generations (n=8). PDE, prenatal dexamethasone exposure, PW, postnatal week. Means ± S.E.M., $^{*}P<0.05$, $^{**}P<0.01$ *vs.* control.

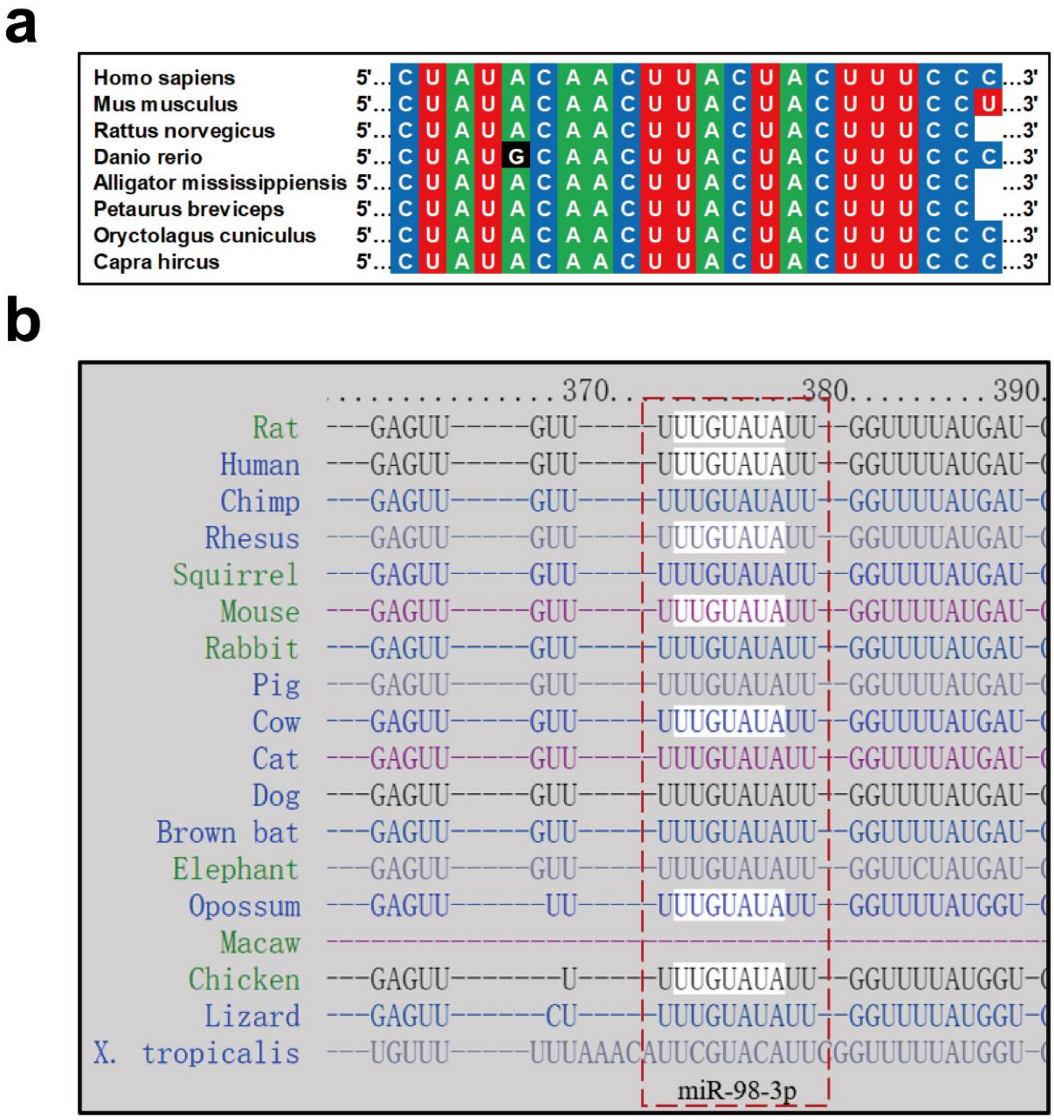

Supplementary Fig. 3 Conservation analysis of miR-98-3p. a. Analysis of miR-98-3p conservation in multiple species. **b.** Conservation analysis of miR-98-3p binding site in the JAG1 3’ -UTR among vertebrates.

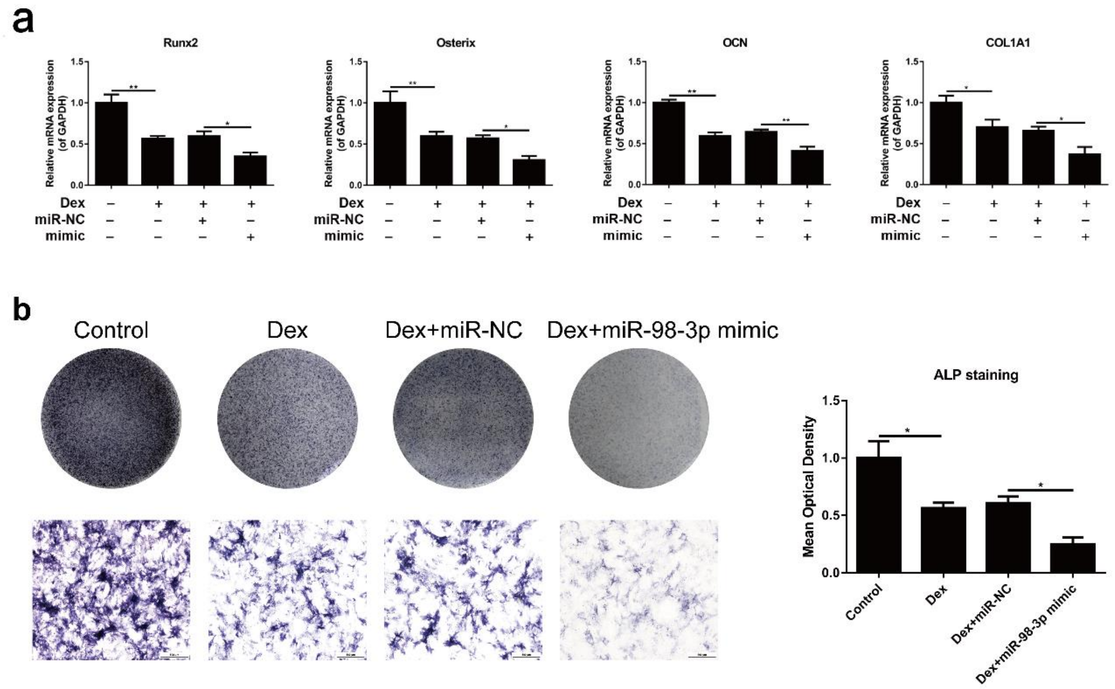

Supplementary Fig. 4 Effects of miR-98-3p mimic on osteogenic differentiation of BMSCs. a. RT-qPCR was used to analyze the expression of osteogenic differentiation marker genes in BMSCs transfected with miR-98-3p mimic under 500 nM dexamethasone and osteogenic differentiation conditions. **b.** ALP staining was performed on day 7 of osteogenic differentiation. Runx2, Runt-related transcription factor 2; OCN，osteocalcin; COL1A1, α1 chain of type I collagen gene; Dex, dexamethasone; miR-NC, miRNA negative control. Means±S.E.M., n=3. $^{*}P<0.05$, $^{**}P<0.01$ *vs.* respective controls.

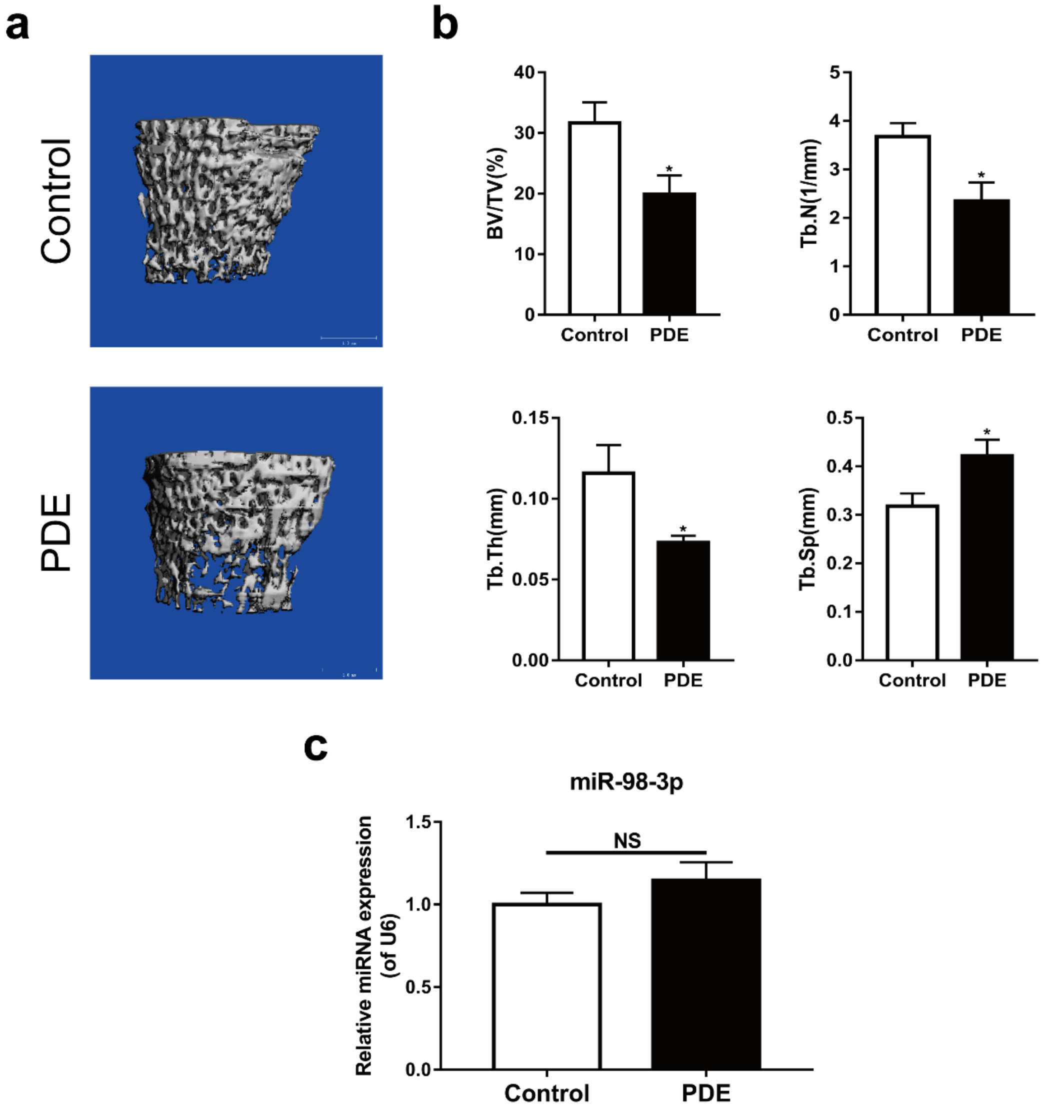

Supplementary Fig. 5 Effects of PDE on bone mass and miR-98-3p expression in F3 male rats. a. Representative micro-CT images of the femur in 8-week-old male rats of the F3 generation (scale bar=1000 μm). **b.** Quantitative micro-CT analysis of trabecular bone microarchitecture. **c.** The expression level of miR-98-3p in long bones of F3 male rats. PDE, prenatal dexamethasone exposure; BV/TV, bone volume/trabecular volume; Tb.N, trabecular number; Tb.Th, trabecular thickness; Tb.Sp, trabecular separation; NS, no significant difference. Means ± S.E.M., n=8. $^{*}P$<0.05 *vs*. control.

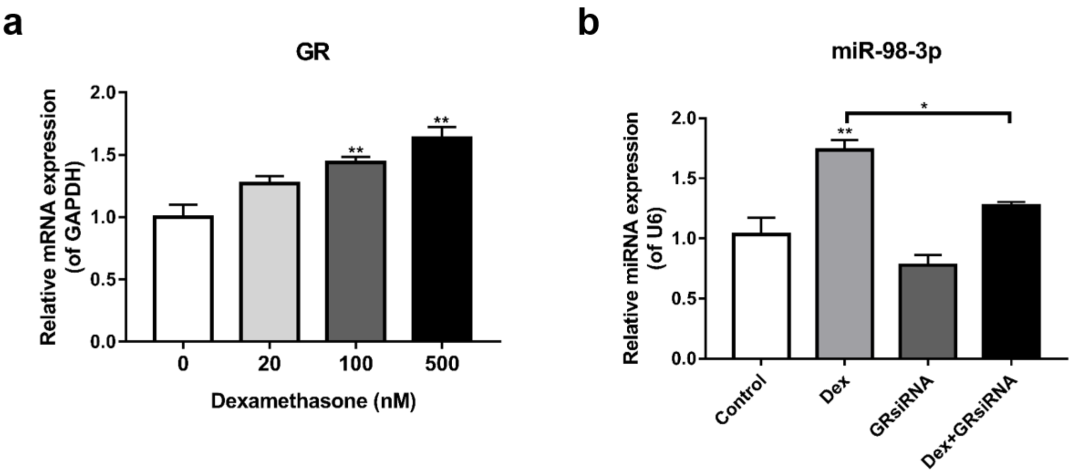

Supplementary Fig. 6 GR mediated the promoting effect of dexamethasone on miR-98-3p expression. a. The mRNA expression level of GR after treating BMSCs with different concentrations of dexamethasone. **b.** The expression level of miR-98-3p after treatment with GR-siRNA. GR, glucocorticoid receptor; Dex, dexamethasone. Mean±S.E.M., n=3. $^{*}P$<0.05, $^{**}P$<0.01 *vs.* respective controls.
